# The Fracture Resistance Comparison between Titanium and Zirconia Implant Abutments with and without Ageing: Systematic Review and Meta-Analysis

**DOI:** 10.3390/dj12090274

**Published:** 2024-08-23

**Authors:** Marek Chmielewski, Wojciech Dąbrowski, Iwona Ordyniec-Kwaśnica

**Affiliations:** 1 SmileClinic Krzysztof Chmielewski, 80-280 Gdansk, Poland; 2 Department of Dental Prosthetics, Faculty of Dentistry, Medical University of Gdansk, 80-210 Gdansk, Poland; wojciech.dabrowski@gumed.edu.pl (W.D.); iwona.ordyniec-kwasnica@gumed.edu.pl (I.O.-K.)

**Keywords:** dental materials, implant abutments, restorative dentistry, material strength, implant prosthetics, zirconia

## Abstract

Implant abutments are essential components of implant prosthetic restorations. The golden standard for abutment material is titanium; however, due to its properties, the esthetic result can be compromised. The most popular esthetic material alternatives are one- and two-piece zirconia. The study aimed to answer the questions of whether zirconia abutments can be used interchangeably with titanium in both anterior and posterior regions and how aging of the abutment affects durability. For this study, an electronic search of MEDLINE (PubMed) and Scopus (Embase) was conducted. The PRISMA guidelines were followed, and a systematic review was registered with PROSPERO. The search revealed 4031 results, of which 17 studies were selected. The strongest material for abutments is titanium, closely followed by two-piece zirconia. One-piece zirconia abutments were the weakest. The cyclic loading above 1,000,000 cycles decreased the fracture resistance of the abutments. Differences in implant diameter, angulation, and restoration affected the fracture strength of all compared materials. The main mode of failure for titanium abutments was screw bending or screw fracture. One-piece zirconia most often presented catastrophic failure with internal hexagon fracture below the implant neck. Two-piece zirconia exhibits a combination of failure modes. Two-piece zirconia abutments may be suitable for use in the posterior region, given their comparable fracture resistance to titanium abutments. Despite the fact that one-piece zirconia is capable of withstanding forces that exceed those exerted during mastication, it is recommended that it be employed primarily in the anterior dentition due to its propensity for unfavorable failure modes.

## 1. Introduction

Restorative treatment utilizing dental implants has become a common procedure in modern dentistry. The implants can be the base for both single and multiunit restorations. The fundamentals for the implant function have been presented in the form of osseointegration by Branemark [1,2]. The advancements in materials and protocols influence the constant inflow of new components, and the multitude of available implant systems generates a diversified array of prefabricated and custom elements with the use of software, which enables ease of case design. Out of all the components used for the restoration, the abutment is one of the most important. There are various abutment types available on the market. Prefabricated abutments have strict dimensions and angulations, which vary depending on the manufacturer. They are meticulously crafted in pristine conditions that won’t affect the properties of the material they are produced from and are easily available. For years, manufacturers have offered prefabricated abutments of various heights and widths. However, prefabricated components sometimes do not deliver enough variety regarding angulation, height, width, and emergence profile to suit the patient’s case. Custom abutments are either manufactured in a dental laboratory by milling the pre-cursors or produced to order. They are designed on an individual basis for each patient. The bite force in the anterior region is estimated to be between 30 to 160 N, while in posterior forces can exceed 400 N [3]. Those values are dependent on muscle strength and mouth opening. Through electromyography (EMG), Gay et al. estimate the bite force to be between 2.5 and 40 kg [4]; thus, abutments need to be durable enough to withstand those forces. Implant abutments are usually produced from titanium. It is considered a golden standard due to its material strength and biocompatibility. However, titanium abutments, especially when used in thin biotypes, can give gingival tissues a blue or grey hue, which is easily visible in the anterior region [5,6]. The alternatives to titanium allowing for immaculate esthetic are alumina, zirconia, polyetheretherketone (PEEK), and polyetherketoneketone (PEKK). The global adoption of computer-aided design/computer-aided manufacturing (CAD/CAM) systems and the increasing accuracy of milling machines have further refined prosthetic designs and fitment of esthetic abutments [7,8,9,10,11]. Zirconia has steadily become a viable option for implant abutments in the anterior region, rivaling titanium due to its excellent mechanical characteristics, marginal accuracy, and internal fit. Like titanium, zirconia also presents good biocompatibility and low plaque retention [11]. Biocompatibility is an especially important material feature, as it enables tight soft tissue seal creation [12]. The abutments have two distinctive areas that encounter soft tissues: the inner layer (around 40 μm), in which fibroblasts adhere directly to the bone and abutments surface (bone-attached), and the outer layer (160 μm) consisting of mainly collagen fibers attached only to the abutment (abutment attached gingiva) [13,14]. Creating a stable soft tissue seal is achievable both in titanium and zirconia abutments and is vital for maintaining peri-implant tissue health and a stable emergence profile of the restorations. Furthermore, when compared with titanium, the use of zirconia abutments leads to a statistically more natural color of soft tissues. This effect is beneficial, particularly in patients with a thin biotype [15]. Before the materials can be used as abutments in dental practice, they need to meet strict conditions in terms of biocompatibility and mechanical durability. From the restoration perspective, the most important mechanical property is load-bearing capability, as it directly influences the durability of planned prosthetic restoration. The fracture strength is most tested using static load to fracture tests with precise control of the force applied and crosshead speed to the abutment. Despite the fact that this method gives accurate results, it doesn’t mimic the masticatory function abutment that will be presented in the oral cavity. Although this can be adjusted by the introduction of cyclic loading in standardized conditions (UNI EN ISO 14801:2016) [16], many studies have started to deviate from them and introduce other cycles to gain a perspective of fatigue influence on abutment material resistance changes. However, this makes the comparisons of such tests difficult. Thermocycling in fatigue testing is especially important in zirconia abutments due to the phenomenon of low-temperature degradation (LTD), which is closely related to Phase Transformation Toughening (PTT). This results in the worsening of mechanical properties until the possible occurrence of spontaneous fractures, even without mechanical stress present [10,17]. The primary aim of this study was to assess the influence of aging on fracture resistance of zirconia abutments and compare the fracture resistance of zirconia abutments pre- and post-aging with the golden standard, the titanium abutments with and without restorations. The secondary aim was to compare the modes of failure of both abutment materials to highlight potential difficulties a clinician may face if the fracture of the abutment occurs. The tertiary aim was to check how restoration material can influence the fracture resistance of the abutments.

## 2. Materials and Methods

The study was registered PROSPERO (International Prospective Register of Systematic Reviews) registry for systematic reviews and granted a number: CRD42023449640.

### 2.1. Search Strategy

**Figure 1:**
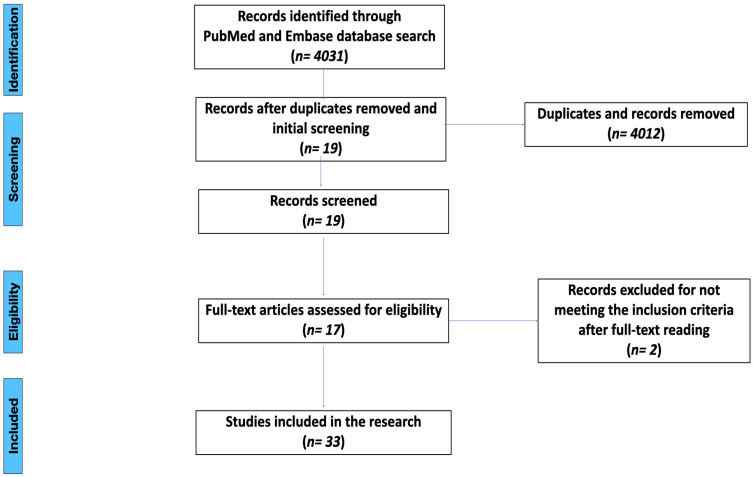
PRISMA flow chart.

An electronic search of MEDLINE (PubMed) and Scopus (Embase) ranging from 1 January 2007 to 29 January 2023 was conducted, covering the last 15 years of studies. The search was done in English language only. The PRISMA (Preferred Reporting Items for Systematic Reviews and Meta-Analyses) guidelines were used [18]. The search terms were used as follows: ‘titanium and zirconia abutments after aging’, ‘zirconia abutments after aging’, ‘titanium abutment’, ‘zirconia abutment’, ‘titanium abutment fracture’, ‘zirconia and titanium fracture resistance’, and ‘zirconia abutment fracture’. The search count is shown in the Figure 1.

### 2.2. Inclusion/Exclusion Criteria

The focused questions for this systematic review were “Do modern zirconia abutments (one-piece and two-piece) provide sufficient resistance to occlusal forces, both in anterior and posterior regions, compared with a titanium control” and “How does abutment aging affect the resistance of zirconia (one-piece and two-piece) and titanium abutments?”.

The articles used in the systematic review reported on in vitro load testing of dental implant abutments and stated the connection type of implant abutment and mode of fracture. The included articles compared titanium with alternative abutment materials; however, there must have been one-piece and/or two-piece zirconia abutments present in the comparison. Articles with and without restorations were included. All considered articles were published in English. Exclusion criteria were: (i) studies not in English, (ii) studies with additional modifications done to zirconia and/or titanium, (iii) studies with a limited description of procedures and materials, (iv) systematic reviews and long-term clinical follow-ups, and (v) studies other than in vitro.

### 2.3. Selection of Studies

The initial search was conducted with a total of 4031 results found. The selection process was performed according to the inclusion criteria. After an initial search, the duplicates were removed, and screening of abstracts was done to meet the inclusion criteria. After abstract screening, 19 articles met the inclusion criteria. Full texts of abstracts meeting the criteria were obtained and evaluated. Overall, 17 relevant articles were considered, 12 of which used the aging procedure before failure testing. The studies selected are shown in Table 1, Table 2, Table 3 and Table 4.

### 2.4. Data Extraction

The data extracted consisted of general article info (Author, Journal, Publisher, and Publish date), general materials info (implant type, implant-abutment connection type, type of restoration, and material of restoration), and specific testing data (methods of fracture testing, methods of aging, measured forces and modes of failure). Gathered data and variables were then put into tables using Excel sheets. If any variable could not be extracted or calculated, they were marked as “not reported”.

### 2.5. Statistical Analysis

Statistical analysis was performed using the STATISTICA 13.3 (TIBCO, Palo Alto, CA, USA). The results for meta-analysis were divided into groups: overall fracture strength, fracture strength dependent on implant diameter, fracture strength with and without aging, fracture strength dependent on the number of cycles during cyclic loading, fracture strength dependent on angulation of indenter, fracture strength during cyclic loading, effect of film addition for stress distribution, fracture strength depending on the crown material. The ANOVA test for independent measures was applied to assess the significance of the extracted results. The level of significance was set to 0.05.

## 3. Results

### 3.1. Implant Dimensions

The study groups considered in this systematic review were largely heterogeneous in terms of width, length and manufacturer of the implant. As the length of an implant has little effect on the fracture resistance of the restoration, different implant diameters and manufacturer designs have a bigger influence on the implant’s neck diameter and surface of the–implant connection. Inadequate diameter can affect the strength and life expectancy of the abutment and accelerate marginal bone loss [35]. Most of the studies were done on 3.7–4.3 mm implants (84%) with internal hexagon connection (88.2%), thus allowing for comparisons between studies.

### 3.2. Testing Parameters

Each study has begun with an appropriate implant fixture. The material of choice was either autopolimeryzing resin [13,19,20,21,22,23,24,25,26,27,28], replicating the modulus of elasticity of human bone, or metal jig [29,30,31,32,33,34]. Out of the 17 considered studies, 12 (70.6%) did not replicate marginal bone loss, stating the implant coverage up to the last thread or not reporting the marginal bone loss simulation. The abutments were torqued according to the manufacturer’s recommendations. The torque values were used according to the manufacturer’s guidelines and ranged between 25–40 Ncm. The restorations used were either ceramic or metal, simulating the upper central incisor. Two studies used three-unit fixed dental prostheses that mimicked upper central and lateral incisors [25,28]. All prostheses and crowns were cemented on the abutments. Three studies did not use any crowns [30,32,33]. After the preparations, specimens were put into testing machine chambers. The forces were applied to the abutments by chromium or stainless-steel indenters in different testing machines. Some of the studies proceeded with fracture resistance testing and fatigue testing.

### 3.3. Cyclic Loading and Thermocycling

There have been thirteen studies that have carried out fatigue testing [13,19,20,21,22,23,24,25,26,28,29,33,34], eight of which proceeded with thermocycling [20,21,22,24,25,26,27,29]. During fatigue testing, eight studies [13,19,21,22,24,28,33,34] kept the specimens in wet conditions (distilled water [13,19,21,24,28] and artificial saline [22,33,34], whereas five [20,23,25,26,29] kept the specimens dry. This, however, did not affect the survival rate of the abutments in any of the compared groups as failures happened both in wet and dry environments. Most of the specimens were loaded at a 30° angle [13,19,20,22,25,28,33,34], although there were studies that placed the specimens at angles of 45° [23], 130° [24] and 135° [21,29]. The loading forces the units were subjected to varied between 40–400 N with frequency of 1.4–2 Hz. The cyclic loading was done at room temperature apart from five cases where cyclic loading was done simultaneously with thermocycling at 5–55 °C [21,23,24,28] or to simulate the oral cavity at 37 °C [34]. During the thermocycling procedure, the temperature was closely monitored in all the studies that have done the procedure [19,20,21,23,24,25,26,28] and ranged between 5–55 °C with intervals between temperature changes of 20–60 s. The number of cycles during fatigue testing ranged between 120,000 to 2,000,000 cycles. In seven studies, the number of cycles was equal to or above 1,200,000 (58.3% of the studies that conducted fatigue testing).

### 3.4. Fracture Load Testing

The specimens were placed at 30° [13,19,25,27,28,30,31,32,34], 45° [23], 90° [32], 130° [24,26] and 135° [21,29]. The testing was carried out in a dry environment at room temperature. Three studies performed cyclic loading and fracture resistance testing simultaneously, achieving fracture during cycling loading [20,22,33]. The results of these three studies were then evaluated separately from conventional load to fracture test due to incomparable methods of testing. The crosshead speed of the fracture testing machine indenter varied between 0.1–2 mm/min, with nine (75%) studies reporting a crosshead speed of 1 mm/min or above [13,21,22,24,25,27,28,34]. Two of the studies did not report the crosshead speed of the indenter [30,33]. Furthermore, there have been seven studies that additionally used Mylar film (0.1 mm thickness) [31] or tin foil (0.5–1 mm thickness) [13,24,25,27,28] to achieve more homogenous pressure distribution area, mimicking the area of antagonist tooth pressure.

### 3.5. Implant Diameter Influence on Fracture Strength

In the considered studies, the implant diameter varied. There have been three distinctive groups created with implant diameters as follows: 3.3–3.8 mm [19,21,25,29,31], 4.0–4.5 mm [13,20,22,23,24,26,27,28,30,31,33,34] and 5.0–5.5 mm [31,32]. The one-piece and two-piece zirconia abutments were compared with titanium abutments both before and after fatigue. The mean values of fracture strengths of titanium abutments before fatigue were 636.78 ±89.52 N for the 3.3–3.8 mm group, 849.2 ± 80.14 N for 4.0–4.5 mm, and 963.65 ± 67.69 N for 5.0–5.5 mm. The difference between the groups was significant (*p* = 0.022676). However, the differences between the groups 4.0–4.5 and 5.0–5.5 mm were not significant (*p* = 0.279987). The mean fracture strength of one-piece zirconia abutments was 483.22 ± 138.79 N for the 3.3–3.8 mm group, 503.83 ± 54.42 N for the 4.0–4.5 mm and 570.04 ± 46.46 N for 5.0–5.5 mm. For two-piece zirconia, mean fracture strengths were 578.19 ± 106.81 N for the 3.3–3.8 mm group, 674.79 ± 80.95 N for 4.0–4.5 mm, and 1286.06 ± 135.58 N for 5.0–5.5 mm. The differences between groups of zirconia abutments were not significant, apart from two-piece zirconia on 5.0–5.5 mm implants, which presented significantly higher fracture strength (*p* < 0.00001 compared to 3.3–3.8 mm group and *p* = 0.000762 compared to 4.0–4.5 mm group). The results of mean fracture values after aging were carried out for 3.3–3.8 mm and 4.0–4.5 mm groups. The titanium abutment’s mean fractures were 800.07 ± 134.7 N for the 3.3–3.8 mm group and 805.06 ± 122.44 N for 4.0–4.5 mm. One-piece zirconia mean fractures were 524.07 ± 73.18 N for 3.3–3.8 mm and 376.4 ± 69.36 N for 4.0–4.5 mm, and two-piece zirconia mean fractures were 861.08 ± 101.05 N for 3.3–3.8 mm and 839 ± 134 N for 4.0–4.5 mm. The differences were not statistically significant.

Further analysis of the results within groups has shown that the least durable abutment is one-piece zirconia. Two-piece zirconia presented better results than one-piece zirconia, sometimes reaching and exceeding the mean fracture values of titanium abutments. All materials presented better fracture strength with the increase of the implant diameter, apart from post-fatigue one-piece zirconia on 4.0–4.5 mm implants. The results of fracture loading between titanium and one-piece zirconia were always statistically significant, unlike two-piece zirconia, where the results reached a significant difference in group 5.0–5.5 mm before fatigue. The results of testing and the significance (*p*-value) are shown in Table 5, Table 6 and Table 7.

### 3.6. Abutment Aging Influence on Fracture Strength

Fracture strengths of abutments were measured before cyclic loading in seven studies [21,24,26,27,30,31,32] and after loading in nine studies [13,19,21,23,24,25,28,29,34], two of which measured fracture strengths before and after cyclic loading [21,24]. The amassed results from the studies were divided into two groups—one without aging and cyclic loading and the other with it. Abutments that have undergone cyclic loading were further divided according to the number of cycles—above or below 1,000,000 cycles.

The mean value for titanium abutments without cycling was 823.03 ± 80.14 N, and with cyclic loading, 803.14 ± 127.88 N, making the difference not significant. One-piece and two-piece zirconia abutments without aging had overall lower mean fracture strength than titanium abutments, with one-piece zirconia averaging 525.09 ± 80.29 N and two-piece zirconia 692.09 ± 94.12 N. The difference was significant only for titanium and one-piece zirconia groups (*p* = 0.000327). After fatigue testing, one-piece zirconia presented lower mean fracture strength than one-piece zirconia without fatigue testing, averaging 412.24 ± 67.4 N (with significant *p* = 0.000028 compared with titanium). However, two-piece zirconia reached 851.62 ± 112.03 N, which is higher than in the group with no aging. The difference between one-piece and two-piece zirconia was significant only in the aging group (*p* = 0.000065).

The increasing number of cycles negatively affected the mean fracture strength of the zirconia abutments, both one-piece (603.58 ± 33.6 vs. 382.08 ± 83.42 N) and two-piece (880.87 ± 70.75 N vs. 839.92 ± 132.68 N). The decrease in fracture strength was significant only for one-piece zirconia abutments (*p* = 0.008352). Unlike titanium, zirconia abutments presented higher fracture strength values in the group with more than 1,000,000 loading cycles. The difference was not significant, though, and the trend inversion may have been due to the low number of titanium abutments exposed to cyclic loading below 1,000,000 cycles. The results of aging influence on fracture strength are shown in Table 5, Table 6, Table 7, Table 8 and Table 9.

### 3.7. Load Angle Influence on Fracture Strength

The articles taken into the meta-analysis used different angles indenters during load-to-fracture testing, including 30°, 45°, 90°, 130°, and 135°. Most of the measurements were done in the 30-degree group as it is the angulation in accordance with ISO guidelines [16]. The groups of 45° and 90° could not have been compared in terms of fatigue, as the 45-degree group only noted results after fatigue, and the 90-degree group noted results without fatigue testing.

The angulation difference before fatigue did not have significance for titanium (*p* = 0.746) and one-piece zirconia (*p* = 0.66). There has been a significant result noted for the two-piece zirconia (*p* = 0.011584). Two-piece zirconia abutments loaded on a 30-degree angle withstood 771.58 ± 77.51 N, whereas those loaded on 130° and 135° withstood 386 ± 116.03 N and 640.45 ± 138.65 N accordingly.

The load-to-fracture values after fatigue testing were significant only for titanium (*p* ≤ 0.00001) abutments. The fracture values after fatigue for one-piece zirconia (*p* = 0.5865) abutments were not significant between different angulation groups. Similarly, the fracture values for two-piece zirconia (*p* = 0.417) abutments were not statistically significant; however, the 130-degree angulation group lacked fatigue load to fracture testing. The results of the loading angle on fracture strength and significance are shown in Table 10, Table 11 and Table 12.

### 3.8. Abutment Material Influence on the Fracture Strength

There have been eight distinctive groups created for the evaluation of the material strength during load-to-fracture testing. They have been as follows: 30° without fatigue, 30° with fatigue, 45° with fatigue, 90° without fatigue, 130° without fatigue, 130° with fatigue, 135° without fatigue, and 135° with fatigue.

In the 30° without fatigue testing group, the highest fracture strength was achieved by titanium abutment with a mean value of 844.38 ± 49.07 N, followed by two-piece zirconia with a mean fracture value of 771.58 ± 77.51 N. One-piece zirconia abutments were the weakest, averaging 558.97 ± 83.96 N. The results for the difference were significant only between titanium and one-piece zirconia (*p* = 0.009012) and reached a *p*-value close to significant between two-piece and one-piece zirconia (*p* = 0.0862). The 30° with fatigue group faired similarly to the group without fatigue testing; however, the highest mean fracture strength has been reported in the two-piece zirconia group with 857.64 ± 154 N, which is higher than without fatigue. Titanium (705.51 ± 62.25 N) and one-piece zirconia (409.15 ± 38.71 N) fracture resistance were lower compared with the group without fatigue testing. The significant results for this group were for titanium and two-piece zirconia compared with one-piece zirconia (*p* = 0.015 and *p* = 0.0025).

The 45-degree group has been tested only after fatigue. The highest mean fracture load has been withstood by titanium abutments (956 ± 168.33 N), followed by two-piece zirconia (568 ± 81 N). The lowest fracture resistance for 45-degree angulation was in the one-piece zirconia group (299 ± 54 N). The statistical analysis regarding the significance of results could not be performed due to the insufficient number of results in the groups.

In the 90-degree angulation, only titanium and one-piece zirconia abutments have been tested. The titanium abutment reached a mean fracture strength of 735.83 ± 87.4 N, whereas one-piece zirconia—299.94 ± 11.94 N. Like the 45-degree group, the statistical significance calculation could not have been performed due to insufficient group sizes.

Another group analyzed was 130° before fatigue testing. Titanium abutment was superior compared to the zirconia abutments, with a mean fracture strength of 765.5 ± 108.76 N. The result for two-piece zirconia was worse than for one-piece zirconia (386 ± 116.03 N vs. 484.67 ± 74.33 N); however, the difference may have been caused by the use of custom-made abutments, not the prefabricated ones. The results were significant for titanium vs. one-piece zirconia (*p* = 0.007), titanium vs. two-piece zirconia (*p* = 0.0022), and one-piece vs. two-piece zirconia (*p* = 0.024). The 130° after fatigue testing group used only titanium and one-piece zirconia abutments. The fracture strengths of both titanium and one-piece zirconia were similar: 484 ± 144 N for titanium and 479.33 ± 89.67 N. The *p*-value could not have been calculated due to the low amount of sample sizes.

The last two groups used 135° of angulation. In the group before fatigue testing, the strongest abutment was made of titanium and withstood, on average, 811.9 ± 171 N of load. Two-piece zirconia abutment followed, averaging 640.45 ± 138.65 N of load. The weakest abutments were made of one-piece zirconia (495 ± 101.4 N). After fatigue, the results were similar, although the abutments in all three material subgroups reached higher average fracture test results. Titanium was the strongest (1075.15 ± 254.05 N), followed by two-piece zirconia (940.13 ± 94.4 N) and one-piece zirconia (516.6 ± 106.57 N). The differences between all three kinds of abutments were significant. The *p*-values were as follows: for titanium and one-piece zirconia, *p* = 0.000148; for titanium and two-piece zirconia, *p* = 0.038457; and for one-piece zirconia and two-piece zirconia, *p* = 0.002681. The results are shown in Table 10, Table 11 and Table 12.

### 3.9. Fracture during Cyclic Loading

There have been studies prepared to check the abutment’s strength during cyclic loading. With each round of simulations, the force of the indenter was increased. Giner et al. [20] used an increasing load ranging from 40 N to 400 N. On the other hand, Cardenas et al. [33] used an increasing load of 88, 170, 210, 250, and 290 N. During such cyclic loading, abutments are fractured under the increasing load. The most durable abutments were two-piece zirconia, which failed at an average of 299 ± 25 N, followed by titanium abutments at 237.2 ± 36.8 N. One-piece zirconia was the least durable, with 158.9 ± 20.75 N on average. The results were significant for titanium and two-piece zirconia when compared with one-piece zirconia, with *p*-values of *p* = 0.006273 and *p* = 0.000217, respectively. The results are in Table 13.

### 3.10. Crown Material Affecting Abutment Fracture Strength

There were different types of restorations put on the abutment for testing. The crowns were either ceramic or metal. The crowns were either prefabricated or custom-made by CAD/CAM. There were also abutments with no crowns used as a control. In the control group without any crown, the strongest abutments were made from zirconia (855.7 ± 107.42 N), followed by titanium (843.51 ± 94.03 N). The weakest abutments were one-piece zirconia (644.54 ± 117.25 N). The differences between groups were not significant. In the test group with ceramic crowns, the strongest were titanium abutments, which withstood, on average, 818.58 ± 114.7 N of force. The strength of two-piece zirconia reduced significantly compared to the control, reaching the mean of 490.58 ± 108.51 N. The weakest abutments were made of one-piece zirconia (417.14 ± 38.55 N). The differences between titanium and both one-piece and two-piece zirconia were significant (*p* = 0.0042 and *p* = 0.01687). In the group utilizing metal crowns, the strongest were titanium (794.29 ± 110.18 N) and two-piece zirconia (762.52 ± 61.03 N) abutments. Again, the weakest abutments were one-piece zirconia (459.96 ± 65.17 N). The results were significant for titanium and two-piece zirconia when compared with one-piece zirconia abutments (*p* = 0.013 and *p* = 0.02428). Results are shown in Table 14.

### 3.11. Abutment Modes of Failure

In all the groups, the most fragile part of an abutment is the implant-abutment connection. In the control group utilizing titanium abutments, the primary mode of failure was screw bending or screw fracture and implant-abutment connection deformation. There have also been prosthetic failures before any significant damage was dealt to the abutment. One-piece zirconia most often presented catastrophic failure with internal hexagon fracture below the implant neck. This results from the brittleness of zirconia and its low resistance to shear forces. Eccentric forces cause the material to deflect, and the connection between the abutment and the implant acts as a breaking edge. Two-piece zirconia presented mixed types of failure due to different materials’ properties. The abutment fracture takes place in the zirconia part, which is located above the implant neck. If the zirconia part withstands the pressure created by the simulator indenter, the mode of failure shifts towards screw bending and plastic deformation of the implant-abutment connection. The most common modes of failure for each abutment material are shown in Table 3.

## 4. Discussion

Replacing lost teeth has often been a challenge. With the introduction of the osseointegration concept [1,36,37] and implantology in dentistry, the need for teeth preparation or removable prosthetics has been in decline. The prosthetics based on implants require an abutment to support the restoration. For many years, the “golden standard” has been the titanium abutments. However, the need for proper esthetic outcomes has grown to be an increasingly important factor in the anterior region, and zirconia abutments have become more popular [38,39]. To predict the behavior of such abutments, preclinical studies were done. By simulating mastication, the failure rate can be assessed, decreasing the chance of implant restoration failure in the patient’s mouth. Furthermore, studies that simulate the maximum load that abutments can handle can indicate whether the abutment is able to handle the masticatory forces of posterior dentition [22]. This aspect is especially important in patients with a wide smile and visible posterior teeth. There is more than one variable that must be taken into account when testing the abutment usability, such as the behavior under different angles, different restorative materials put on the abutment, the dimensions of abutment and implant base, or the persistence of the material toughness over time [40,41,42,43,44,45,46,47,48,49,50]. This systematic review has been created to assess whether fatigue testing significantly affects the abutment strength and to analyze how the fracture strength varies depending on the material and the setup used to measure the abutment resilience.

The cyclic loading test is designed to simulate the masticatory cycle in a preclinical environment [51,52]. The lab method is both cost-efficient and predictable, as the simulator uses constant pressure with constant speed within given cycles. Although the machine setting is similar in the literature, there are other variables that may affect the results. The most important factors seem to be the number of cycles performed, the angle of the indenter, and hydrothermal stress. First and foremost, implant abutments must withstand many loading cycles while maintaining mechanical stability and material properties [53,54,55,56]. The lack of standardization decreases the credibility of the results and makes the comparisons difficult, as each variable can affect the outcome. In that sense, focusing on the cyclic loading tests can result in more clinically relevant results than the basic measurement of fracture strength. The increasing load during fatigue testing mimicking masticatory behavior results in stress cumulation and implant–abutment misfit [57,58], consequently leading to premature abutment failure when compared with static load to fracture [20,22,33,59]. The ISO 14801 standard states the minimal number of cycles (1,000,000 or above), the angulation of the indenter (30°), 2 mm implant neck exposure, and the upper force limit of the indenter (100 N), which in bulk tests are difficult to keep precise [16,60]. A million cycles at 2 Hz corresponds to around 12 months of clinical function [61]. With the ISO 14801 requirement, only seven studies have been complied that reach 1,000,000 or more cycles and only one reached 2,000,000 cycles. Five studies reported less than 1,000,000 cycles. The results have shown that the strength of one-piece zirconia abutments is affected by the number of cycles during fatigue testing.

The problem with heterogeneity followed the fracture strength testing. Different indenters were used between studies (semispherical or round). Furthermore, there were a total of five different angulations used during load to fracture—30°, 45°, 90°, 130°, and 135°. The division according to the angulation has been made to assess how the angulation affects the results. The in-group heterogeneity has also been noticeable, as different implant diameters, number of abutments tested, restoration materials, and artificial aging procedures have been used. It is recommended that future research in this field place greater emphasis on the importance of the guidelines presented by the ISO, to make the results of studies more comparable and easier to interpret. Despite different methodologies used, the fracture values were sufficient for the use of all types of studied abutments in anterior dentition. Two-piece and titanium abutments were similarly matched, indicating the possibility for two-piece zirconia to be the substitute for titanium in patients requiring immaculate esthetics.

## 5. Conclusions

From this systematic review, conclusions can be drawn:

- The implant diameter positively correlates with the fracture strength of abutments.
- Titanium and two-piece zirconia abutments retain similar fracture strength after cyclic loading, while one-piece zirconia abutments show significantly reduced fracture strength after cyclic loading.
- The increase in the number of cycles during fatigue testing negatively affects one-piece zirconia fracture strength.
- Despite different angulations, the fracture strength of abutments stays similar between the groups.
- Two-piece zirconia abutments withstood the most cycles and the highest force during cyclic loading until fracture.
- The use of ceramic cemented crowns noticeably decreases the fracture strength of both one-piece and two-piece zirconia abutments, whereas metal cemented crowns significantly decrease the fracture strength of only one-piece zirconia.

## Figures and Tables

**Table 1:** Studies selected for the systematic review.

	Authors	Journal	Title	Date
1	Elsayed, A., et al. [13]	Clinical Oral Implants Research	Effect of fatigue loading on the fracture strength and failure mode of lithium disilicate and zirconia implant abutments	2018
2	Atsü, S.S., et al. [19]	The International Journal of Oral and Maxillofacial Implants	Fracture Resistance of Titanium, Zirconia and Ceramic-Reinforced Polyetheretherketone Implant Abutments Supporting CAD/CAM Monolithic Lithium Disilicate Ceramic Crowns After Aging	2019
3	Giner, S., et al. [20]	Journal of Prosthetic Dentistry	Fatigue fracture resistance of titanium and chairside CAD-CAM zirconia implant abutments supporting zirconia crowns: An in vitro comparative and finite element analysis study.	2021
4	Alsahhaf, A., et al. [21]	Journal of The Mechanical Behavior of Biomedical Materials	Fracture resistance of zirconia-based implant abutments after artificial long-term aging	2017
5	Foong, J.K., et al. [22]	The Journal of Prosthetic Dentistry	Fracture resistance of titanium and zirconia abutments: an in vitro study	2013
6	Sen, N., et al. [23]	The Journal of Prosthetic Dentistry	Fatigue survival and failure resistance of titanium versus zirconia implant abutments with various connection designs	2019
7	Att, W., et al. [24]	Clinical Implant Dentistry and Related Research	Influence of Preparation and Wall Thickness on the Resistance to Fracture of Zirconia Implant Abutments	2012
8	Saker, S., et al. [25]	International Journal of Oral and Maxillofacial Implants	Fracture Resistance of Straight and Angulated Zirconia Implant Abutments Supporting Anterior Three-Unit Lithium Disilicate Fixed Dental Prostheses	2016
9	Sghaireen, M.G. [26]	Clinical Implant Dentistry and Related Research	Fracture Resistance and Mode of Failure of Ceramic versus Titanium Implant Abutments and Single Implant-Supported Restorations	2015
10	Elsayed, A., et al. [27]	The Journal of Prosthetic Dentistry	Comparison of fracture strength and failure mode of different ceramic implant abutments	2017
11	Karasan, D., et al. [28]	The International Journal of Prosthodontics	Mechanical Stability of Zirconia Implant Abutments Supporting Cantilevered Fixed Dental Prostheses After Fatigue Loading	2021
12	AlAmar, M., et al. [29]	Journal of Oral Implantology	The effect of different implant-abutment connection materials on the fracture resistance of zirconia abutments	2020
13	Watanabe, S., et al. [30]	Materials	Fracture resistance of zirconia abutments with or without a titanium base: an in vitro study for tapered conical connection implants	2022
14	Shabanpour, R., et al. [31]	The Journal of Contemporary Dental Practice	Comparative Evaluation of Fracture Resistance and Mode of Failure of Zirconia and Titanium Abutments with Different Diameters	2015
15	Yang, J., et al. [32]	Clinical Oral Implants Research	Fracture Resistance of inter-joined zirconia abutment of dental implant system with injection molding technique	2013
16	Cárdenas, R., et al. [33]	The Journal of Prosthetic Dentistry	Effect of fatigue loading and failure mode of different ceramic implant abutments	2021
17	Markarian, R.A., et al. [34]	The Journal of Prosthetic Dentistry	Dental Implant-abutment fracture resistance and wear induced by single-unit screw-retained CAD components fabricated by four CAM methods after mechanical cycling	2020

**Table 2:** Methods of testing in studies.

1st Author Date	Implant Type Dimensions	Implant Fixture	Simulated Marginal Bone Level Changes	Implant-abutment Connection	Abutment Torque Value	Specimens in Group	Restoration	Restoration Fixture	Type of Titanium Abutment + Manufacturer	Type of Zirconia Abutment + Manufacturer
Elsayed, A et al., 2018 [13]	FairTwo (FairImplant) 4.2 × 11.5 mm	Autopolimeryzing resin (Technovit 4000)	0 mm	internal hexagon	25 Ncm	8	Lithium-Disilicate (IPS E.max CAD Ivoclar Vivadent)	Cemented (Multilink Automix)	FairImplant titanium abutment (FairImplant)	One-piece and two-piece zirconia (not reported)
Atsü, S.S. et al., 2019 [19]	Sky Implant (Bredent) analogs 3.5 × 9 mm	Autopolimeryzing resin (Technovit 4071)	Not reported	Internal hexagon	25 Ncm	12	Monolithic lithium disilicate	Cemented (Panavia V5)	Grade 4 Ti (Bredent)	Two-piece zirconia (Bredent)
Giner, S., et al., 2021 [20]	Screw line promote plus (Camlog Implants) 4.3 × 11 mm	Epoxy Resin (EpoxiCure 2)	3 mm	Tube-In-Tube	20 Ncm	11	Monolithic zirconia	Cemented (Speed Cem Plus)	Camlog Esthetic	Two-piece zirconia (Incoris ZI CAD/CAM + CAD/CAM titanium base)
Alsahhaf, A., et al., 2017 [21]	SIC Invent 3.3 mm	Autopolimeryzing resin (Technovit 4000)	1.5 mm	Internal hexagon	20 Ncm	16	Metal crown (Kera S-powder)	Cemented (Panavia 21)	Grade 5 Ti (SIC Invent)	One-piece CAD/CAM zirconia (SINA-Z), two-piece zirconia luted (SINA-Z + SIC Invent), two-piece zirconia cemented (SINA-Z + SIC Invent + Panavia 21) and one-piece prefabricated (White Star)
Foong, J.K., et al., 2013 [22]	OsseoSpeed (AstraTech Dental AB) 4 × 9 mm	Autopolimeryzing resin (Unifast Trad III)	0 mm	Internal hexagon	20 Ncm	11	Metal crown (Coron Institute Straumann AB)	Cemented (Panavia F2.0)	TiDesign (AstraTech Dental AB)	One-piece ZirDesign (AstraTech Dental AB)
Sen, N., et al., 2019 [23]	Nobel Parallel CC RP, Replace Select Straight TiU RP, 4.3 × 10 mm NobelSpeedy Groovy RP (Nobel Biocare) 4 × 10 mm	Autopolimeryzing resin (Technovit 4000)	3 mm	Internal conical, internal tri-channel and external hexagonal	35 Ncm	9	Monolithic zirconia (VITA YZ ST)	Cemented (RelyX Unicem 3M ESPE)	Procera Esthetic (Nobel Biocare)	One- and two-piece Esthetic Abutment (Nobel Biocare)
Att, W. et al., 2012 [24]	Nobel Replace Straight Groovy (Nobel Biocare) 4 × 13 mm	Autopolimeryzing resin (Technovit 4000)	Not reported	Internal hexagon	35 Ncm	16	Chromium-cobalt metal crown (Dentitan)	Cemented (Panavia 21)	NobelProcera Titanium RP (Nobel Biocare)	One-piece NobelProcera Zirconia RP (Nobel Biocare)
Saker, S et al., 2016 [25]	Legacy (Implant Direct) 3.7 × 13 mm	Epoxy Resin (System Three Resin)	Not reported	Internal hexagon	30 Ncm	8	Lithium-Disilicate three-unit FDP	Cemented (RelyX Unicem 3M ESPE)	Titanium abutment (Implant Direct)	One-piece Zirconia abutment (Implant Direct)
Sghaireen, MG 2015 [26]	Oraltronics (Oraltronics Dental Implant Technology GmbH) 4 × 10 mm	Autopolimeryzing resin (Melliodent Heraeus Kulzer)	0 mm	internal hexagon	30 Ncm	15	Ti: metal-ceramic (Remanium G-Soft + VITA ceramics) Zr-1: In-Ceram Alumina (VITA) Zr-2: IPS Empress Esthetic (Ivoclar Vivadent)	Cemented (Super Dent Glass Ionomer)	Oraltronics titanium abutments (Oraltronics Dental Implant Technology GmbH)	One-piece and two-piece Oraltronics zirconia abutments (Oraltronics Dental Implant Technology GmbH)
Elsayed, A., et al., 2017 [27]	FairTwo (FairImplant) 4.2 × 11.5 mm	Autopolimeryzing resin (Technovit 4000)	0 mm	internal hexagon	25 Ncm	8	Lithium-Disilicate (IPS E.max CAD Ivoclar Vivadent)	Cemented (Multilink Automix)	FairImplant titanium abutment (FairImplant)	One-piece and two-piece zirconia (not reported)
Karasan, D., et al., 2021 [28]	Bone Level (Strauman AG) 4.1 × 13 mm	Autopolimeryzing resin (Technovit 4000)	not reported	internal hexagon	35 Ncm	8	Two- and three-unit FDP cobalt-chromium (Wirobond C+)	Cemented (Multilink Automix)	Anatomic Ti Abutment (Straumann AG)	One-piece (IPS e.max Anatomic Abutment Straumann)
AlAmar, M., et al., 2020 [29]	Nobel Replace (Nobel) analogs (3.5 mm)	Metal jig	Not reported	Internal hexagon	35 Ncm	7	No restoration	No restoration	Not reported	Not reported
Watanabe, S., et al., 2022 [30]	Nobel Replace (Nobel) 4.3 × 10 mm Roxolid BLT (Straumann) 4.1 × 10 mm	Collet chuck (EY Collet)	0 and 3 mm	Internal hexagon	35 Ncm	3	Hemispherical cap (SK material)	Not reported	Nobel Titanium (Nobel Biocare) and Straumann Titanium (Straumann)	One-piece and two-piece Nobel Procera (Nobel Biocare) and CARES (Straumann)
Shabanpour, R., et al., 2015 [31]	XiVE (Dentsply Friadent) 3.4 mm 3.8 mm 4.5 mm 5.5 mm	Metal jig	Not reported	Internal hexagon	24 Ncm	10	No restoration	NO restoration	XiVE titanium abutment (Dentsply Friadent)	one-piece XiVE, two-piece XiVE (Dentply Friadent), one-piece Zirkonzahn (Zirkonzahn GmbH)
Yang, J et al., 2013 [32]	Osstem GSII (Osstem Implants) 5 × 10 mm	Metal jig	Not reported	Internal hexagon	40 Ncm	10	No restoration	No restoration	Osstem GSII (Osstem Implants)	One-piece Zirconia (TZ-3YB-E Tosh)
Cárdenas, R., et al., 2021 [33]	Bone Level RC (Straumann AG) 4.1 × 11 mm	metal jig	0 mm	internal hexagon	35 Ncm	10	Lithium-Disilicate (IPS E.max Press Ivoclar Vivadent)	Cemented (Multilink Hybrid Abutment)	Variobase Ti (Straumann AG)	One-piece (Straumann CARES) and two-piece (Variobase TiBase)
Markarian, R.A., et al., 2020 [34]	Implant analogs (AN4100 S.I.N) 4.1 mm	metal jig	3 mm	internal hexagon	32 Ncm	10	No restoration	No restoration	AI 4151-Q (S.I.N.)	CAD/CAM abutment (Z-CAD HD)

**Table 3:** Load to fracture testing results with and without aging.

1st Author Date	Titanium Fracture Strength before Fatigue (N)	One-Piece Zirconia Fracture Strength before Fatigue (N)	Two-Piece Zirconia Fracture Strength before Fatigue (N)	Environment	Load Application Axis angle	Indenter	Testing Device	Crosshead Speed	Fracture Titanium Strength after Fatigue (N) ± SD	Fracture One-Piece Zirconia Strength after Fatigue (N) ± SD	Fracture Two-Piece Zirconia Strength after Fatigue (N) ± SD	Primary Mode of Failure of Two-Piece Zirconia	Primary Mode of Failure of One-Piece Zirconia	Primary Mode of Failure of Titanium
Elsayed, A., et al., 2018 [13]	Not reported	Not reported	Not reported	Dry	30°	Semispherical loading stamp	Universal testing machine (Germany 2050, Zuick/Roell) with 0.5 mm tin foil	2 mm/min	900 N	Median 198 N	944 N	Screw fracture and implant connection deformation	Internal hexagon fracture	Screw fracture and implant connection deformation
Atsü, S.S., et al., 2019 [19]	Not reported	Not reported	Not reported	Dry	30°	Spherical loading stamp	Universal testing machine (Lloyd LRK 10 Plus) with 0.5 mm tin foil	0.5 mm/min	787.8 ± 120.9 N	Not reported	623.93 ± 121 N	Screw fracture and implant connection deformation	Not reported	Abutment and crown fracture
Giner, S., et al., 2021 [20]	Not reported	Not reported	Not reported	Dry	30°	Universal indenter	Electromagnetic testing machine (Shimadzu EMT series EMT-1kn-30; Shimadzu)	Not reported	240 N at 2 × 10^6^ cycles	Not reported	340 N at 2 × 10^6^ cycles	Screw bending and implant neck fracture	Not reported	Screw bending and implant neck fracture
Alsahhaf, A., et al., 2017 [21]	811.8 ± 171 N	CAD/CAM: 509.5 N ± 117.6 N Prefabricated: 480.5 ± 85.2 N	Cemented: 660.2 ± 140.3 N Luted: 620.7 ± 137 N	Dry	135°	Semispherical loading stamp	Universal testing machine (Zwick Z010/TN2S)	2 mm/min	1054.8 ± 217.6 N	CAD/CAM: 228.8 N ± 172.5 N Prefabricated: 580.8 ± 92.6 N	Cemented: 833.5 ± 104.4 N Luted: 849.1 ± 158.3 N	Screw fracture and implant connection deformation	Internal hexagon fracture	Screw fracture and implant connection deformation
Foong, J.K., et al., 2013 [22]	Not reported	Not reported	Not reported	Wet (artificial saline)	30°	Rounded metal loading platen	Closed-loop servo hydraulics (MTS 810 Materials Test System) with graphite	Not reported	269.6 ± 56.7 N	139.8 ± 24.6 N	Not reported	Screw fracture and implant connection deformation	Internal hexagon fracture	Not reported
Sen, N., et al., 2019 [23]	Not reported	Not reported	Not reported	Dry	45°	Spherical loading stamp	Universal testing machine (Shimadzu AG-IS)	1 mm/min	ICT: 1069 ± 182 N ITT: 926 ±197 N EHT: 873 ± 126 N	ICZ: 287 ± 63 N EHZ: 311 ± 45 N	ITZ: 568 ± 81 N	Abutment fracture and screw/abutment plastic deformation	Fracture below implant shoulder ICZ fracture above implant shoulder EHZ	Plastic deformation of screw and abutment
Att, W., et al., 2012 [24]	519 ± 85 N	Zr-1: 493 ± 73 N Zr-8: 488 ± 82 N Zr-18: 473 ± 68 N	Not reported	Dry	130°	Semispherical loading stamp	Universal testing machine (Zwick Z010/TN2S) with 1 mm tin foil	2 mm/min	484 ± 144 N	Zr-1: 481± 66 N Zr-8: 479 ± 101 N Zr-18: 478 ± 102 N	Not reported	Not reported	Zr-1: primary mode unable to identify Zr-8: screw bending Zr-18: abutment fracture	Screw bending
Saker, S., et al., 2016 [25]	Not reported	Not reported	Not reported	Dry	30°	Universal indenter	Universal testing machine (Lloyd) with 1 mm tin foil	1 mm/min	Ti15: 523.57 ± 19.71 N Ti0: 538.7 ± 24.77 N	Zr15:528.37 ± 24.57 N Zr0: 542.17 ± 21.64 N	Not reported	Not reported	Not reported–FDP broke first	Not reported–FDP broke first
Sghaireen, M.G., 2015 [26]	1012 ± 132.51 N	Not reported	Zr1: 498 ± 154.98 N (Alumina crown) Zr2: 274 ± 77.07 N (IPS Empress Esthetic crown)	Dry	130°	Universal indenter	Universal testing machine (Instron 1195)	1 mm/min	Not reported	Not reported	Not reported	Crown and abutment fracture	Not reported	Screw bending
Elsayed, A., et al., 2017 [27]	900 N	218.5 ± 14.4 N	900 N	Dry	30°	Semispherical loading stamp	Universal testing machine (Germany 2050, Zuick/Roell) with 0.5 mm tin foil	2 mm/min	Not reported	Not reported	Not reported	Screw bending	Internal hexagon fracture	Screw bending
Karasan, D., et al., 2021 [28]	Not reported	Not reported	Not reported	Dry	30°	Universal indenter	Universal testing machine (Germany 2050, Zuick/Roell) with 0.5 mm tin foil	1 mm/min	Ti-1:601 ±41.51 N Ti-2: 664.5 ±37.59 N	Zr-1: 226 ± 26.45 N Zr-2: 551.2± 82.19 N	Not reported	Not reported	Internal hexagon fracture above implant shoulder	Plastic deformation of implant-abutment connection
AlAmar, M., et al., 2020 [29]	Not reported	Not reported	Not reported	Dry	135°	Universal indenter	Universal testing machine (not reported)	Not reported	1095.5 ± 290.5 N	740.2 ± 54.6 N	1137.8 ± 20.5 N	Not reported	Not reported	Not reported
Watanabe, S., et al., 2022 [30]	NB 0 mm: 1050 N ST 0 mm: 1100 N NB 3 mm: 600 N ST 3 mm: 800 N	NB 0 mm:459.9 ± 13.2 N ST 0 mm: 693.9 ± 37.2 N NB 3 mm: 383.8 ± 7.9 N ST 3 mm: 551.2 ± 15.8 N	NB 0 mm: 507.3 ±22 N ST 0 mm: 1142.7± 36.9 N NB 3 mm: 425.6 ± 30.3 N ST 3 mm: 827.9 ± 14.3 N	Dry	30°	Universal indenter	Universal testing machine (Electro Puls E3000)	0.5 mm/min	Not reported	Not reported	Not reported	Zirconia fracture near titanium base and titanium base deformation	Internal hexagon fracture	Screw bending
Shabanpour, R., et al., 2015 [31]	Ti 3.4: 597.96 ± 54.35 N Ti 3.8: 500.59 ± 43.21 N Ti 4.5: 740.08 ± 32.12 N Ti 5.5: 1120.02 ± 52.01 N	Zr 3.8: 688.48 ± 109.47 N Zr 4.5: 838.99 ± 99.62 N ZrCAD 3.4: 286.41 ± 257.08 N ZrCAD 3.8: 451.21 ± 124.61 N ZrCAD 4.5: 725.04 ± 200.78 N ZrCAD 5.5: 989.54 ± 98.22 N	ZTi 3.4: 605.08 ± 63.64 N ZTi 3.8: 426.79 ± 86.29 N ZTi 4.5: 822.79 ± 231.09 N ZTi 5.5: 1286.06 ± 135.58 N	Dry	30°	Universal indenter	Universal testing machine (Germany 2050, Zuick/Roell) with 0.1 mm Mylar film	0.1 mm/min	Not reported	Not reported	Not reported	Screw fracture and body fracture	Internal hexagon fracture	Screw fracture
Yang, J., et al., 2013 [32]	B30: 1035.1 ± 63.66 N B90: 735.83 ± 87.4 N	A30: 420.63 ± 29.22 N A90: 299.94 ± 11.94 N	Not reported	Dry	90°, 30°	Universal indenter	Universal testing machine (AGS-J)	Not reported	Not reported	Not reported	Not reported	Not reported	Not reported	Not reported
Cárdenas, R., et al., 2021 [33]	Not reported	Not reported	Not reported	wet (Artificial saline)	30°	Universal indenter	Chewing Simulator (UANL FIME)	Not reported	202 ± 16.9 N at 25.8 k ± 5.5 k cycles	178 ± 16.9 N at 13.5 k ± 7 k cycles	258 ± 25 N at 48.2 k ± 7.4 k cycles	Crown and abutment fracture	Internal hexagon fracture	Screw fracture and implant connection deformation
Markarian, R.A., et al., 2020 [34]	Not reported	Not reported	Not reported	Dry	30°	Universal indenter	Universal testing machine (Emic DL-2000)	1 mm/min	923 + 129 N	Not reported	1005 ± 187 N	Not reported	Abutment fracture	Screw bending and Implant destruction

**Table 4:** Fatigue cyclic loading and thermocycling testing.

1st Author Date	Simulator	Angle	Environment	Temperature	Force (N)	Number of Cycles	Frequency	Thermocycling
Elsayed, A., et al., 2018 [13]	Chewing Simulator (CS-4 SD Mechatronik GmbH)	30°	Wet (distilled water)	room temperature	49 N	1.2 × 10^6^	1.6 Hz	Not reported
Atsü, S.S., et al., 2019 [19]	Chewing Simulator (CS-4 SD Mechatronik GmbH)	30°	Wet (distilled water)	Room temperature	100 N	4.8 × 10^5^	1.6 Hz	5–55 ± 5 °C in distilled water for 30 s during 2000 cycles
Giner, S., et al., 2021 [20]	electromagnetic testing machine (Shimadzu EMT series EMT-1kn-30; Shimadzu)	30°	Dry	Room temperature	40–400 N	2 × 10^6^	2 Hz	5–55 ± 5 °C in artificial saliva for 20 s during 10 000 cycles
Alsahhaf, A., et al., 2017 [21]	Artificial chewing simulator (Willytec)	135°	Wet (distilled water)	5–55 °C	49 N	1.2 × 10^6^	Not reported	5–55 °C in distilled water for 60 s during cyclic loading
Foong, J.K., et al., 2013 [22]	closed-loop servo hydraulics (MTS 810 Materials Test System)	30°	Wet (artificial saline)	Room temperature	100, 150, 200, 250, 300 and 400 N	2 × 10^4^ at each force threshold	2–5 Hz	Not reported
Sen, N., et al., 2019 [23]	occlusal loading-chewing simulator (Esetron Smart Robotechnologies)	45°	Dry	5–55 °C	50 N	1.2 × 10^6^	2 Hz	5–55 °C for 60 s 5000 times
Att, W., et al., 2012 [24]	Artificial chewing simulator (Willytec)	130°	Wet (distilled water)	5–55 °C	49 N	1.2 × 10^6^	1.6 Hz	5–55 °C for 60 s during whole cyclic loading
Saker, S., et al., 2016 [25]	Load cycling device (not reported)	30°	Dry	Room temperature	50 N	6 × 10^5^	2 Hz	5–55 ± 2 °C for 20 s during 6000 cycles
Sghaireen, M.G., 2015 [26]	not reported	Not reported	Dry	Not reported	Not reported	Not reported	Not reported	5–55 °C for 30 s during 3000 cycles
Elsayed, A., et al., 2017 [27]	not reported	Not reported	Not reported	Not reported	Not reported	Not reported	Not reported	Not reported
Karasan, D., et al., 2021 [28]	Artificial chewing simulator (C.S 4.8, Willytec)	30°	wet	5–55 °C	49 N	1.2 × 10^6^	1.6 Hz	5–55 °C for 60 s during whole cyclic loading
AlAmar, M., et al., 2020 [29]	Artificial chewing simulator (not reported)	135°	Dry	Room temperature	Not reported	2.5 × 10^5^	Not reported	Not reported
Watanabe, S., et al., 2022 [30]	not reported	Not reported	Not reported	Not reported	Not reported	Not reported	Not reported	Not reported
Shabanpour, R., et al., 2015 [31]	not reported	Not reported	Not reported	Not reported	Not reported	Not reported	Not reported	Not reported
Yang, J., et al., 2013 [32]	not reported	Not reported	Not reported	Not reported	Not reported	Not reported	Not reported	Not reported
Cárdenas, R., et al., 2021 [33]	Chewing Simulator (UANL FIME)	30°	Wet (artificial saline)	room temperature	88, 170, 210, 250, 290 N	1 × 10^5^	1.4 Hz	Not reported
Markarian, R.A., et al., 2020 [34]	Load cycling device (Bicycle Biopdi)	30°	Wet (artificial saline)	37 °C	100 N	1 × 10^6^	2 Hz	Not reported

**Table 5:** Mean fracture strength values and significance of material on fracture strength when compared with titanium control.

Implant Diameter	3.3–3.8 mm without Fatigue Testing	3.3–3.8 mm with Fatigue Testing	4–4.5 mm without Fatigue Testing	4–4.5 mm with Fatigue Testing	5–5.5 mm without Fatigue Testing
Average material fracture strength	Titanium: **636.78 ± 89.52 N** One-piece: **483.2 ± 138.79 N** Two-piece: **578.19 ± 106.81 N**	Titanium: **800.07 ± 134.70 N** One-piece: **524.07 ± 73.18 N** Two-piece: **861.08 ± 101.05 N**	Titanium: **849.20 ± 80.14 N** One-piece: **503.83 ± 54.42 N** Two-piece: **674.79 ± 80.95 N**	Titanium: **805.06 ± 122.44 N** One-piece: **376.40 ± 69.38 N ** Two-piece: **839.00 ± 134.00 N**	Titanium: **963.65 ± 67.69 N** One-piece: **570.04 ± 46.46 N ** Two-piece: **1286.06 ± 135.58 N**
Fracture resistance vs. titanium control (significant if *p* < 0.05)	One-piece zirconia: **0.096734** Two-piece zirconia: 0.688568	One-piece zirconia: **0.098033** Two-piece zirconia: 0.89824	One-piece zirconia: **0.000371** Two-piece zirconia: 0.153244	One-piece zirconia: **0.000181** Two-piece zirconia: **0.7573**	One-piece zirconia: **0.018037** Two-piece zirconia: **0.000987**
Fracture resistance one-piece vs. two-piece zirconia (significant if *p* < 0.05)	**0.020068**	**0.07323**	**0.11934**	**0.000191**	**0.000275**

**Table 6:** Significance of implant diameter on abutment fracture strength.

	Titanium before Fatigue	Titanium after Fatigue	One-Piece Zirconia before Fatigue	One-Piece Zirconia after Fatigue	Two-Piece Zirconia before Fatigue	Two-Piece Zirconia after Fatigue
Implant 3–3.8 mm vs. 4–4.5 mm (significant if *p* < 0.05)	**0.049276**	**0.970114**	**0.8222**	**0.124293**	**0.49341**	**0.865513.**
Implant 3–3.8 mm vs. 5–5.5 mm (significant if *p* < 0.05)	**0.003834**	**No data**	**0.53225**	**No data**	**<0.00001**	**No data**
Implant 4–4.5 mm vs. 5–5.5 mm (significant if *p* < 0.05)	**0.279987**	**No data**	**0.549308**	**No data**	**0.000762**	**No data**

**Table 7:** Significance of fatigue testing on abutment fracture strength.

Material	Titanium	One-Piece Zirconia	Two-Piece Zirconia
*p*-value with vs. without fatigue testing (significant if *p* < 0.05)	3.3–3.8 mm: **0.2507 ** 4–4.5 mm: **0.648392 ** 5–5.5 mm: **No data**	3.3–3.8 mm: **0.707122 ** 4–4.5 mm: **0.10327 ** 5–5.5 mm: **No data**	3.3–3.8 mm: **0.014531 ** 4–4.5 mm: **0.280685 ** 5–5.5 mm: **No data**

**Table 8:** Mean fracture resistance with and without aging regardless of implant diameter.

	Titanium without Aging	One-Piece Zirconia without Aging	Two-Piece Zirconia without Aging	Titanium with Aging	One-Piece Zirconia with Aging	Two-Piece Zirconia with Aging
Fracture (N)	**823.03 ± 80.14**	**525.09 ± 80.29**	**692.09 ± 94.12**	**803.14 ± 127.88**	**412.24 ± 67.40**	**851.62 ± 112.03**
Fracture with aging below 10^6^ (N)	**No data**	**No data**	**No data**	**736.39 ± 113.97**	**603.58 ± 33.60**	**880.87 ± 70.75**
Fracture with aging above 10^6^ (N)	**No data**	**No data**	**No data**	**832.81 ± 134.34**	**382.08 ± 83.42**	**839.92 ± 132.68**

**Table 9:** Significance of aging on fracture strength.

	Titanium:	One-Piece Zirconia:	Two-Piece Zirconia:
*p*-value compared with titanium without aging (significant if *p* < 0.05)		**0.000327**	**0.197881**
*p*-value compared with titanium with aging (significant if *p* < 0.05)		**0.000028**	**0.633917**
*p*-value one-piece compared with two-piece zirconia with aging (significant if *p* < 0.05)		**0.000065**	
*p*-value one-piece compared with two-piece zirconia without aging (significant if *p* < 0.05)		**0.067789**	
*p*-value non aged compared with aged abutments (significant if *p* < 0.05)	**0.434193**	**0.008352**	**0.720565**

**Table 10:** Mean fracture strength in Newtons (N) depending on angulation.

	Titanium: (N)	One-Piece Zirconia: (N)	Two-Piece Zirconia: (N)
30° before fatigue	**844.38 ± 49.07**	**558.97 ± 83.96**	**771.58 ± 77.51**
30° after fatigue	**705.51 ± 62.25**	**409.15 ± 38.71**	**857.64 ± 154.00**
45° after fatigue	**956.00 ± 168.33**	**299.00 ± 54.00**	**568.00 ± 81.00**
90° before fatigue	**735.83 ± 87.4**	**299.94 ± 11.94**	**No data**
130° before fatigue	**765.50 ± 108.76**	**484.67 ± 74.33**	**386.00 ± 116.03**
130° after fatigue	**484.00 ± 144.00**	**479.33 ± 89.67**	**No data**
135° before fatugue	**811.80 ± 171.00**	**495.00 ± 101.40**	**640.45 ± 138.65**
135° after fatigue	**1075.15 ± 254.05**	**516.60 ± 106.57**	**940.13 ± 94.40**

**Table 11:** Significance of angulation on fracture strength.

	30° before Fatigue	30° after Fatigue	130° before Fatigue	135° after Fatigue
*p*-value titanium vs. one-piece zirconia (significant if *p* < 0.05)	**0.009012**	**0.014695**	**0.006987**	**0.000148**
*p*-value titanium vs. two-piece zirconia (significant if *p* < 0.05)	**0.56445**	**0.130836**	**0.00219**	**0.038457**
*p*-value one-piece vs. two-piece zirconia (significant if *p* < 0.05)	**0.086192**	**0.002493**	**0.024151**	**0.002681**

**Table 12:** Significance of angulation and fatigue on fracture strength.

	30°	130°
Titanium abutment *p*-value before vs. after fatigue (significant if *p* < 0.05)	**0.192624**	**0.006869**
One-piece abutment zirconia *p*-value before vs. after fatigue (significant if *p* < 0.05)	**0.79677**	**0.10928**
Two-piece abutment zirocnia *p*-value before vs. after fatigue (significant if *p* < 0.05)	**0.571616**	**No data**

**Table 13:** Mean fracture during fatigue testing.

	Titanium:	One-Piece Zirconia:	Two-Piece Zirconia:
Fracture (N)	**237.2 ± 36.8**	**158.9 ± 20.75**	**299 ± 25**
*p*-value compared with titanium control (significant if *p* < 0.05)		**0.006273**	**<** **0.00001**
*p*-value one-piece compared with two-piece zirconia (significant if *p* < 0.05)		**0.000217**	

**Table 14:** Fracture resistance (N) depending on restoration type and significance.

	Titanium:	One-Piece Zirconia:	Two-Piece Zirconia:
Ceramic (N)	**818.58 ± 114.7**	**417.14 ± 38.55**	**490.98 ± 108.51**
Metal (N)	**794.29 ± 110.18**	**459.96 ± 65.17**	**762.52 ± 61.03**
No restoration (N)	**843.51 ± 94.03**	**644.54 ± 117.25**	**855.7 ± 107.42**
*p*-value different crown and same abutment material (significant if *p*< 0.05)	**0.914465**	**0.064686**	**0.115534**
*p*-value ceramic compared with titanium (significant if *p*< 0.05)		**0.00420**	**0.01687**
*p*-value metal compared with titanium (significant if *p*< 0.05)		**0.01298**	**0.95775**
*p*-value no restoration compared with titanium (significant if *p*< 0.05)		**0.38550**	**0.99624**
*p*-value ceramic one-piece zirconia compared with two-piece zirconia (significant if *p*< 0.05)		**0.75641**	
*p*-value metal one-piece zirconia compared with two-piece zirconia (significant if *p*< 0.05)		**0.02428**	
*p*-value no crown one-piece zirconia compared with two-piece zirconia (significant if *p*< 0.05)		**0.34404**	

## Data Availability

The original contributions presented in the study are included in the article.
